# Role of Intravenous Azithromycin as Adjunctive Therapy in Children With Acute Encephalitis Syndrome (AES): An Open-Label Randomized Controlled Trial

**DOI:** 10.7759/cureus.87387

**Published:** 2025-07-06

**Authors:** Priti Yadav, Kundan Mittal, Paramjeet S Gill, Anindya Mittal, Kausalya Raghuraman, Jaya S Kaushik

**Affiliations:** 1 Pediatrics, Pandit Bhagwat Dayal Sharma Post Graduate Institute of Medical Sciences, Rohtak, IND; 2 Microbiology, Pandit Bhagwat Dayal Sharma Post Graduate Institute of Medical Sciences, Rohtak, IND; 3 Critical Care Medicine, Pandit Bhagwat Dayal Sharma Post Graduate Institute of Medical Sciences, Rohtak, IND; 4 Microbiology, All India Institute of Medical Sciences, Guwahati, IND; 5 Pediatrics, All India Institute of Medical Sciences, Guwahati, IND

**Keywords:** acute encephalitis, azithromycin, cerebrospinal fluid, iv ceftriaxone, pediatric scrub typhus

## Abstract

Background: Acute encephalitis syndrome (AES) has high morbidity and mortality in children. Empirical treatment of AES often consists of third-generation cephalosporins, with vancomycin and acyclovir, and frequently excludes the use of azithromycin, targeted at scrub typhus. In a resource-constrained setting, testing for scrub typhus becomes challenging. Considering the lacunae in the existing literature, there is a need for robust evidence to determine the role of additional azithromycin use in children with AES.

Objective: To evaluate the efficacy and safety of adjunctive intravenous azithromycin treatment in children with AES compared to standard therapy alone.

Material and methods: An open-label, two-arm randomized controlled trial was conducted at a tertiary care teaching hospital with a level III pediatric intensive care unit. Children aged one to 14 years with AES were enrolled. Intravenous azithromycin in addition to conventional treatment (n=30) and conventional treatment (third-generation cephalosporin, vancomycin, and acyclovir) were compared. The primary outcome measure was all-cause mortality. Secondary outcome measures included the total length of hospital stay, the proportion of children with significant disability as determined by the Liverpool Outcome Score (LOS) at discharge, and the proportion of children who experienced at least one serious adverse event.

Results: The two groups were comparable in terms of all-cause mortality (23.3% vs. 20%; p=0.75), duration of hospital stay (12.57 days vs. 13.67 days; P=0.28), and significant disability at the time of discharge (52.40 vs. 53.47 days; P=0.79). None developed serious life-threatening adverse events.

Conclusion: Additional treatment with intravenous azithromycin does not impact all-cause mortality among children with AES. Further evaluation is suggested with an adequately powered study and long-term follow-up.

## Introduction

Acute encephalitis syndrome (AES) is an important cause of mortality and morbidity in children in India. The causative agent of AES varies with season and geographical location and predominantly affects the population below 15 years of age. There have been several outbreaks of AES in Uttar Pradesh with an incidence of 15 per million population and a case fatality rate of 12.6% in the years 2015-2019. One Rickettsial infection contributes to 0.5-2% of AES in Uttar Pradesh [1]. Presumptive treatment of febrile illnesses with doxycycline and azithromycin has been associated with a reduction in the progression of febrile illness to AES in the Gorakhpur district of Uttar Pradesh [2]. Although some studies have examined the use of azithromycin in preventing the progression from acute febrile illness to AES, there are no studies evaluating the empirical use of azithromycin for the treatment of AES. Beyond Uttar Pradesh, there are emerging reports of frequent scrub typhus outbreaks in other parts of India, including Rajasthan, Haryana, Meghalaya, and even islands in the Pacific Ocean. In an adult study from Haryana, scrub typhus was detected in 16% of patients admitted with fever lasting more than seven days [3]. AES caused by scrub typhus is treatable with doxycycline and/or azithromycin, which can significantly reduce mortality. The standard management protocol for AES often includes a combination of third-generation cephalosporins, vancomycin, acyclovir, and/or artesunate [4]. However, there is limited literature on the effectiveness of adding azithromycin to the existing treatment regimen for AES. Therefore, this study was planned to evaluate the efficacy and safety of azithromycin in the management of AES in children.

## Materials and methods

The study was an open-label, two-arm, randomized controlled trial conducted in the Department of Pediatrics and Microbiology at Pandit Bhagwat Dayal Institute of Medical Sciences, Rohtak, Haryana, a tertiary care referral center in India. The study extended from April 2020 to March 2021. The study was reported in accordance with CONSORT guidelines. Children aged one to 14 years with suspected AES were enrolled in the study. AES was considered in the acute onset of fever with change in the mental status like confusion, disorientation, or coma, and/or new onset of seizure (excluding simple febrile seizure). Recruitment of patients commenced after obtaining ethical approval from the Biomedical Research Ethics Committee (IEC/Th/19/Ped04 dated 30th December 2019). Informed written consent was obtained from the parents of all enrolled subjects, and a patient information sheet was provided to them before enrollment in the study. The study was registered with CTRI (CTRI/2020/05/025270) before the enrollment of the first patient.

All eligible participants were admitted to the pediatric intensive care unit. A detailed clinical history and physical examination were performed in each child. All children were enrolled sequentially and allotted a study number. They were randomized by block randomization using variable block sizes of two, four, and six, generated by computer using random number tables, into two groups: an interventional group (azithromycin (intravenous 10 mg/kg for five days) plus conventional treatment) and a control group (conventional treatment). Azithromycin (500 mg/vial) was used and prepared by diluting 4.8 mL sterile water in a vial to generate a concentration of 100 mg/mL. A sealed opaque envelope containing group codes was prepared. Envelops were sequentially numbered and kept in order according to their serial numbers. The envelope was opened at the time of randomization, and the patient was allocated to their respective group.

Patients were closely monitored for the need for mechanical ventilation, inotropic support, and signs of multi-organ dysfunction. Five milliliters of blood were collected for ELISA testing for scrub typhus, HIV serology, and thick and thin peripheral smears for malaria parasites. Patients were investigated and managed as per the standard treatment protocol. Any adverse event following drug administration was recorded. The primary outcome measure was all-cause mortality. The secondary outcomes include the total duration of hospital stay and the Liverpool outcome score (LOS). The LOS (open access) was performed at the time of discharge, and the mean score was computed to assess the degree of disability [5].

A convenience sample size of 30 participants was chosen for each group, considering logistical limitations. All the measurements and data were analyzed using conventional statistical tools. All data was entered in Microsoft Excel (Microsoft Corporation, Redmond, WA) spreadsheets. Normally distributed variables were described by their mean and standard deviation. For normally distributed data, the Student t-test was employed, and the Mann-Whitney and Chi-square tests were used for categorical data. All data were analyzed using IBM SPSS Statistics (Version 20.0; IBM Corp., Armonk, NY, USA).

## Results

**Figure 1:**
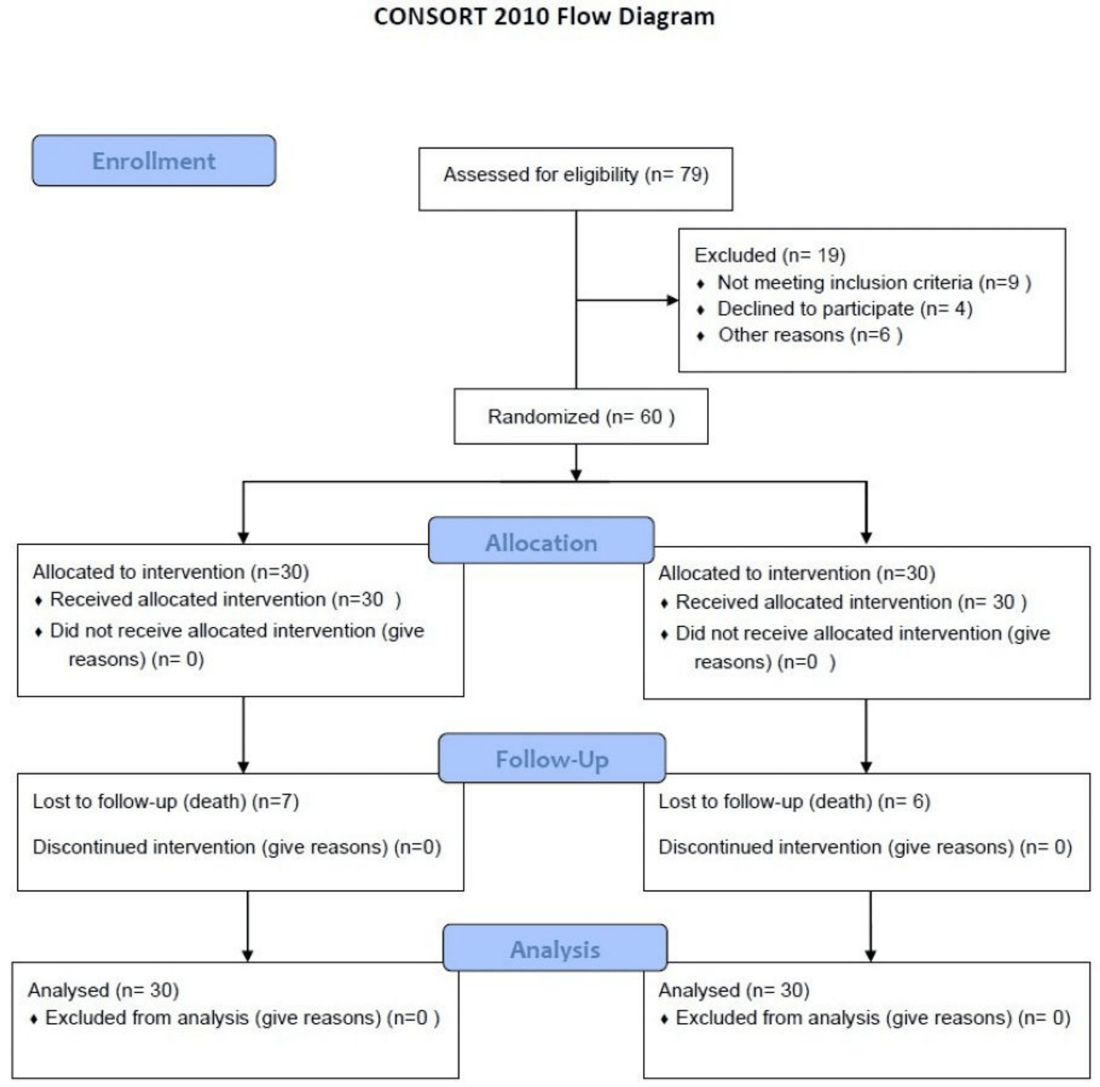
Study flow Figure showing the enrollment of study subjects.

Out of 79 eligible participants, 60 children were randomized, 30 to receive azithromycin and 30 to receive conventional treatment (Figure 1).

The baseline demographic characteristics were comparable between the two groups (Table 1). The majority of children presented with fever, abnormal body movement, and altered sensorium in both groups (Table 1).

**Table 1:** Baseline comparison of enrolled participants P-values were determined using the Student's t-test* and the chi-square test (for all other comparisons). CSF: cerebrospinal fluid; ELISA: enzyme-linked immunosorbent assay; HPF: high power field

Characteristics	Azithromycin group	Conventional treatment group	P-value
Demographics			
Age (mean (SD))	7.37 (3.82)	6.10 (3.84)	0.18*
Male gender (n (%))	13 (43.3%)	17 (56.7%)	0.31
Rural residence	16 (53.3%)	23 (76.7%)	0.06
Urban residence	14 (46.7%)	7 (23.3%)	0.06
Clinical features			
Fever	30 (100.0%)	30 (100.0%)	1
Abnormal body movements	30 (100.0%)	28 (93.3%)	0.49
Altered sensorium	30 (100.0%)	30 (100.0%)	1
Rash	0 (0.0%)	3 (10.0%)	0.24
Headache	5 (16.7%)	6 (20.0%)	0.74
Nausea/vomiting	9 (30.0%)	9 (30.0%)	1
Arthralgia	2 (6.7%)	2 (6.7%)	1
Myalgia	1 (3.3%)	1 (3.3%)	1
Loose stools	10 (33.3%)	7 (23.3%)	0.39
Examination			
Pallor (yes)	19 (63.3%)	18 (60.0%)	0.79
Hepatomegaly	5 (16.7%)	5 (16.7%)	1
Hepatosplenomegaly	0 (0.0%)	1 (3.3%)	1
Investigations			
ELISA for scrub typhus	1 (3.3%)	1 (3.3%)	1
CSF: protein (mg/dL)	80.80±36.94	106.50±64.85	0.08*
CSF: sugar (mg/dL)	70.00±21.20	72.20±39.34	0.69*
CSF: cells (/HPF)	23.17±46.27	27.70±65.39	0.36*

Only one child in each group tested positive for scrub typhus. On comparison of the primary outcome measure, all-cause mortality was comparable between the azithromycin group (7 (23.3%)) and the conventional treatment group (6 (20%)) (P=0.75). Secondary outcome measures, including duration of hospital stay (12.57 vs. 13.67 days; P=0.28) and LOS (52 vs. 53.4; P=0.79), were also comparable between the two groups (Table 2).

**Table 2:** Outcome measure of enrolled participants P-values were determined using the chi-square test* and Student's t-test^.

Outcome measure	Azithromycin group (n=30)	Conventional treatment group (n=30)	P-value
All-cause mortality (N (%))	7 (23.3%)	6 (20.0%)	0.75*
Duration of hospital stay in days (mean (SD))	12.57 (10.5)	13.67 (6.8)	0.28^
Liverpool outcome score at discharge (Mean (SD))	52.4 (28.14)	53.47 (27.78)	0.79^

Only one child in the azithromycin group developed a rash and another developed hypotension.

## Discussion

Preliminary findings of this study suggest that the addition of azithromycin provided no extra benefit in children with AES in terms of all-cause mortality, duration of hospital stay, or significant disability at discharge. The side effect profile was comparable between the two groups.

Over the past decade, there has been a paradigm shift in the etiology of AES, from Japanese encephalitis (JE) to other infectious and non-infectious causes. Scrub typhus has emerged as a significant cause of AES in North India [6-9]. In a study on 464 children with acute encephalitis, 2.9% of children died, with 6.7% having neurological disability [6]. The case fatality rate varies considerably, and districts of Assam have reported 27.6% mortality among children with AES [10]. In a study from South India, 23% of children with AES had scrub typhus [11]. Authors reported a sensitivity of 93% and a specificity of 82% for IgM when compared to PCR, as used in our study.

In several districts of Uttar Pradesh, the case fatality rate declined to 5-5.8% in 2019-2020, compared to 33% in the early 1980s. Only 0.5-2.0% of cases were attributed to Rickettsial infection. There are several reports of scrub typhus as an emerging cause of AES [12-14].

Authors attribute this sharp decline to aggressive immunization campaigns for JE and presumptive treatment of febrile cases with doxycycline and azithromycin [15-17]. The present study observed a mortality rate of 20-23.3%. The comparable mortality in the present study could be attributed to only two cases that tested positive for scrub typhus on serum testing. In the present study, the additional use of azithromycin did not affect mortality, despite its more extensive coverage, which not only targets Rickettsial infections but also covers Mycoplasma infections. The present study provides evidence on the additional use of azithromycin among children with AES, demonstrating its neutral effect on mortality, duration of hospital stay, and neuro-morbidity. 

The present study had certain limitations, including a small sample size, and the interventions were not masked. A facility for testing all neuroviruses in cerebrospinal fluid (CSF) was one of the logistical limitations, and only CSF JE antibodies were tested. There could be many more confounding factors, such as differences in CSF protein, delay in seeking treatment, enrollment during a particular season, or inclusion of a migrating population, which will need to be considered when interpreting the results of the present study. Estimating the entire neuroviral panel would have helped establish the etiology of AES. Moreover, neuroimaging and EEG could not be performed on all enrolled children due to limitations. A lack of long-term follow-up of patients is one of the limitations of the present study. However, authors believe that in the majority of peripheral centers, facilities for neuroimaging, EEG, and long-term follow-up are lacking.

In light of the above limitations, the present study provides some preliminary evidence to discourage routine use of azithromycin among children with AES. However, further studies with a larger sample size and long-term follow-up will be required before limiting the use of azithromycin in the management of AES among children.

## Conclusions

In this study with a limited sample size, the addition of azithromycin provided no benefit among children with AES in terms of all-cause mortality, duration of hospital stay, or significant disability at discharge. Only two cases of scrub typhus were identified, for which azithromycin is considered the drug of choice in children. Therefore, we conclude that adjunctive treatment with intravenous azithromycin did not impact all-cause mortality in children with AES in our study. However, a larger sample size and a multicentric study are needed to better understand the effect of azithromycin on all-cause mortality in children with AES.

## References

[1] Venkatesan A, Tunkel AR, Bloch KC (2013). Case definitions, diagnostic algorithms, and priorities in encephalitis: consensus statement of the International Encephalitis Consortium. Clin Infect Dis.

[2] Sharma S, Mishra D, Aneja S, Kumar R, Jain A, Vashishtha VM (2012). Consensus guidelines on evaluation and management of suspected acute viral encephalitis in children in India. Indian Pediatr.

[3] Joshi R, Kalantri SP, Reingold A, Colford JM Jr (2012). Changing landscape of acute encephalitis syndrome in India: a systematic review. Natl Med J India.

[4] Ghosh S, Basu A (2016). Acute encephalitis syndrome in India: the changing scenario. Ann Neurosci.

[5] Lewthwaite P, Begum A, Ooi MH (2010). Disability after encephalitis: development and validation of a new outcome score. Bull World Health Organ.

[6] Murhekar MV, Mittal M, Prakash JA (2016). Acute encephalitis syndrome in Gorakhpur, Uttar Pradesh, India - role of scrub typhus. J Infect.

[7] Mittal M, Bondre V, Murhekar M (2018). Acute encephalitis syndrome in Gorakhpur, Uttar Pradesh, 2016: clinical and laboratory findings. Pediatr Infect Dis J.

[8] Mittal M, Thangaraj JW, Rose W (2017). Scrub typhus as a cause of acute encephalitis syndrome, Gorakhpur, Uttar Pradesh, India. Emerg Infect Dis.

[9] Vivian Thangaraj JW, Mittal M, Verghese VP (2017). Scrub typhus as an etiology of acute febrile illness in Gorakhpur, Uttar Pradesh, India, 2016. Am J Trop Med Hyg.

[10] Khan SA, Bora T, Laskar B, Khan AM, Dutta P (2017). Scrub typhus leading to acute encephalitis syndrome, Assam, India. Emerg Infect Dis.

[11] Damodar T, Singh B, Prabhu N (2023). Association of scrub typhus in children with acute encephalitis syndrome and meningoencephalitis, southern India. Emerg Infect Dis.

[12] Husain U, Arpita Arpita, Kalyan RK (2023). An alarming surge of scrub typhus cases presenting as acute encephalitis in children. Trop Doct.

[13] Murhekar MV (2017). Acute encephalitis syndrome and scrub typhus in India. Emerg Infect Dis.

[14] Kar A, Dhanaraj M, Dedeepiya D, Harikrishna K (2014). Acute encephalitis syndrome following scrub typhus infection. Indian J Crit Care Med.

[15] Fang Y, Huang Z, Tu C, Zhang L, Ye D, Zhu BP (2012). Meta-analysis of drug treatment for scrub typhus in Asia. Intern Med.

[16] Jang MO, Jang HC, Kim UJ (2014). Outcome of intravenous azithromycin therapy in patients with complicated scrub typhus compared with that of doxycycline therapy using propensity-matched analysis. Antimicrob Agents Chemother.

[17] Kim YS, Yun HJ, Shim SK, Koo SH, Kim SY, Kim S (2004). A comparative trial of a single dose of azithromycin versus doxycycline for the treatment of mild scrub typhus. Clin Infect Dis.

